# *Pterospoda nigrescens* (Hulst), a synonym of *Ixala klotsi* Sperry (Lepidoptera, Geometridae, Ennominae)

**DOI:** 10.3897/zookeys.149.2343

**Published:** 2011-11-24

**Authors:** Clifford D. Ferris, B. Christian Schmidt

**Affiliations:** 1 5405 Bill Nye Ave., R.R. 3, Laramie, WY 82070, USA. Research Associate: McGuire Center for Lepidoptera and Biodiversity, Florida Museum of Natural History, University of Florida, Gainesville, FL; C. P. Gillette Museum of Arthropod Diversity, Colorado State University, Ft. Collins, CO; Florida State Collection of Arthropods, Gainesville, FL; 2 Canadian Food Inspection Agency, Canadian National Collection of Insects, Arachnids, and Nematodes, KW Neatby Bldg., 960 Carling Ave., Ottawa, Ontario, Canada K1A 0C6

**Keywords:** Arizona, Caberini, Ennominae, Geometridae, *Ixala*, Lepidoptera, Mexico, nomenclature, *Pterospoda*, taxonomy, Texas

## Abstract

Comparison of the types of *Ixala klotsi* (Sperry) and *Pterospoda nigrescens* (Hulst) shows that they are the same species, with *Ixala klotsi* a synonym of *Pterospoda nigrescens*. A lectotype of *Selidosema nigrescens* is designated, and the types of *Selidosema nigrescens* and *Ixala klotsi* are illustrated. Male and female habitus and genitalia of *Pterospoda nigrescens* are illustrated.

## Introduction

Hulst (1898) described *Selidosema nigrescens* based on an unknown number of female specimens from San Antonio, Texas. Dyar (1903) subsequently placed *Selidosema nigrescens* in *Pterospoda*. Sperry (1940) described *Ixala klotsi* from two males and a female that he and his wife collected in the Baboquivari Mountains, Pima County, Arizona. We have examined the type material of both taxa and compared contemporary specimens from Texas and Arizona to each other and to the types, and conclude that both names refer to the same species. The purpose of this note is to synonymize *Ixala klotsi* under *Selidosema nigrescens*, designate a lectotype for the latter, and discuss the generic concepts in the *Pterospoda* group of genera.

Repository abbreviations:

AMNH American Museum of Natural History, New York, NY, USA


CNC Canadian National Collection of Insects, Arachnids, and Nematodes, Ottawa, Ontario, Canada


CDF Personal collection of Clifford D. Ferris, Laramie, Wyoming, USA


## Systematics

### Pterospoda nigrescens

(Hulst)

http://species-id.net/wiki/Pterospoda_nigrescens

Selidosema nigrescens Hulst, 1898:214.
Ixala klotsi Sperry, 1940: 146. syn. n.

#### Type material.

**Figures 1–7.**
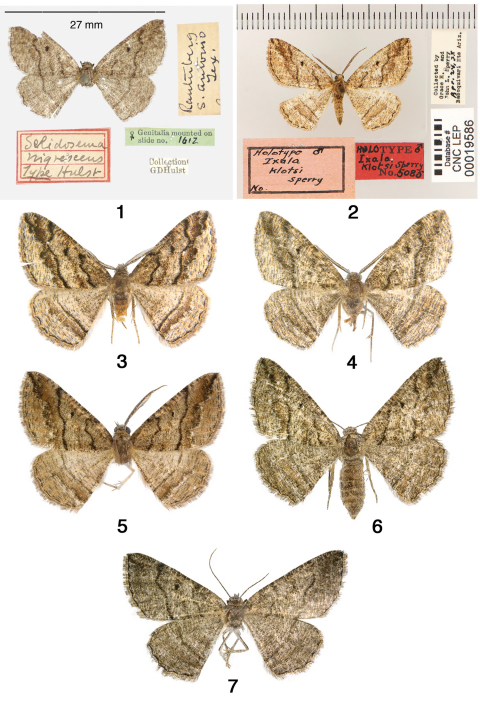
*Pterospoda nigrescens*. **1** Lectotype of *Pterospoda nigrescen*s **2** Holotype of *Ixala klotsi* **3** male AZ Cochise Co. **4** male AZ Pima Co. **5** male TX Val Verde Co. **6** female AZ Santa Cruz Co. **7** female TX Brewster Co.

*Selidosema nigrescens*. Female lectotype [here designated], Texas, [Bexar Co.], San Antonio [AMNH] (Fig. 1). A label for the lectotype will be added as follows: “Lectotype / *Selidosema / nigrescens* Hulst, 1898 / Ferris & Schmidt 2011.” *Ixala klotsi*. Male holotype, Arizona, [Pima Co.], Baboquivari Mts. 24 April, 1938, Grace H. and John L. Sperry [CNC] (Fig. 2).

#### Other material examined.

**USA.** **Arizona.** Cochise Co.: Forest Road 42B, Coronado Nat. For., 1525m , 19.viii.1981, C. D. Ferris (1m); Guadalupe Canyon, 8.viii.1979, C. D. Ferris (1m); Gray Hawk Nature Preserve, 1235 m, 10.ix.2010, C. D. Ferris (1f). Pima Co.: Baboquivari Mtns., 15. iv [no year], S.E. Cassino (2m); Baboquivari Mts., Brown Canyon, 1183 m, 18.viii.2006, C. D. Ferris (1m), 27.vii.2007, C. D. Ferris (8m, 2f); Baboquivari Mtns., Brown Cyn., 22.iv.2006, B. Walsh (3m); 10.vii.2005 (1m); 2.viii.2008, B. C. Schmidt (2m); base of Tortolita Mts., 888 m, all C. D. Ferris, 4–8.iv.2003 (1m, 1f), 27-28.ix.2003 (2m, 2f), 24.x.2003 (1f), 4.x.2004 (1f), 4–11.iv.2005 (1m, 1f), 24.x.05 (1m), 9.x.2006 (1f). Santa Cruz Co., all C. D. Ferris. Patagonia, 1235 m, 8.viii.2009 (1f); Peña Blanca Canyon, 1200 m, 8.ix.2010 (6m, 1f). **Texas.** Brewster Co. Green Gulch, Big Bend N. P., 6–10.ix.2008 (1f), B[ordelon]/K[nudson]. Sinton Co., Welder Refuge 4.iv.1981 (1f). Uvalde Co., Concan, Neal’s Lodges, 9.iv.1990, N. McFarland (1m, 1f); Concan, 25.iii.1985, E. Knudson (1m). Val Verde Co., Del Rio, 25.iv.1959, M.R. MacKay (1m, 1f), 4.x.1994 (1m), E. Knudson. **MEXICO.** Durango: 25 mi. W. of Durango, 7500’ 6.v.1961, H. Howden & J.E.H. Martin (1m); 10 mi. W. of Durango, 7500’, 15.v.1964, W.C. McGuffin (1m, 1f); 5 mi. W. of Durango, 6500’ 11.vi.964, J.E.H. Martin (1f).

#### Discussion.

The type specimen of *Selidosema nigrescens* in AMNH does not bear a holotype label. Hulst stated in his description: “...all the specimens before me are females.” The specimen label data agree with Hulst’s description, and we therefore consider this specimen to be a syntype. The locations of the other syntypes are unknown. In order to ensure stability of the name, we designate this specimen as the lectotype (Fig. 1). The holotype of *Ixala klotsi* and additional specimens of *Pterospoda nigrescens* are illustrated in Figs 1–7.

**Figures 8–13.**
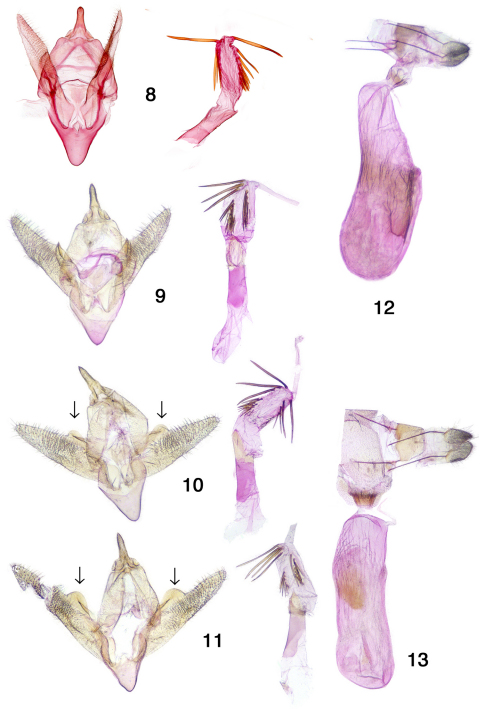
*Pterospoda nigrescens* genitalia. **8** genitalia of **1** **9** genitalia of **4** **10** male AZ Pima Co. **11** genitalia of **5** **12** female AZ Pima Co. **13** genitalia of **7**

Genitalia structure (Figs. 8–13). The orientation and degree of flattening of the male genital capsule produces different aspects. The natural position is shown for the holotype of *Ixala klots*i (Fig. 8). A partially flattened preparation is shown in Fig. 9, and strongly flattened preparations in Figs 10–11, in which the approximately semicircular projections (indicated by arrows) at the base of the costa become evident. The number and positions of the spines on the everted vesicae are somewhat variable, suggesting that the spines are partially deciduous. The balsam genitalia slide of the female lectotype of *Pterospoda nigrescens* was made in 1950 without use of stains, and the tissues were strongly cleared. There is very little contrast between the preparation and the now yellowed balsam, and we were unable to obtain a photograph suitable for publication. The form of the genitalia is consistent with specimens from Texas (Fig. 13) and Arizona (Fig. 12). The corpus bursae of the Texas specimen (Fig.13) appears slightly smaller than that of the Arizona specimen (Fig. 12). Upon dissection, it was found to be only partially expanded, and was mechanically manipulated into the position shown.

*Ixala* Hulst and *Pterospoda* Dyar were placed in the Caberini along with 11 other genera by Ferguson (1983), the most recent tribal classification for this primarily North American tribe. Only *Pterospoda* and *Sericosema* Warren enter the Neotropical region in Mexico (Pitkin 2002). Rindge (1949) revised the *Drepanulatrix* group of genera, but the remaining genera in Caberini are in need of revision. For example, preliminary data suggest that both *Ixala* and *Pterospoda* as currently defined are not natural groups; *Pterospoda opuscularia* (Hulst), the type-species of *Pterospoda*, is more closely related to *Apodrepanulatrix litaria* (Hulst), and the remaining species in *Ixala* and *Pterospoda* have little in common in both facies and genitalic structure.

*Pterospoda nigrescens* occurs at moderate elevations in arid scrub and open woodland habitat, ranging from south-eastern Arizona and the Edwards Plateau of west-central Texas south to at least Durango, Mexico (Fig. 14). It is not known from New Mexico, but should occur there. Collection dates indicate at least two annual flights, primarily in April and August in Arizona. In Pima Co., AZ, Ray Nagle (Tucson, AZ) has reared the moth on a *Condalia* species, probably *Condalia warnockii* var. *kearneyana* M. C. Johnson (Rhamnaceae).

**Figure:**
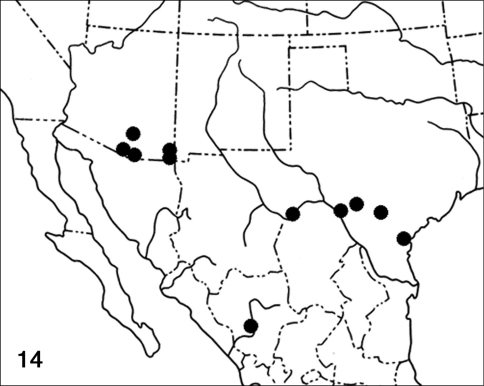
**Figure 14.** Distribution map for *Pterospoda nigrescen*s.

## Supplementary Material

XML Treatment for Pterospoda nigrescens
